# hTERT promotes cell adhesion and migration independent of telomerase activity

**DOI:** 10.1038/srep22886

**Published:** 2016-03-14

**Authors:** Haiying Liu, Qianqian Liu, Yuanlong Ge, Qi Zhao, Xiaohui Zheng, Yong Zhao

**Affiliations:** 1 Key Laboratory of Gene Engineering of the Ministry of Education, School of Life Sciences, Sun Yat-sen University, Guangzhou 510006 P. R. China; 2 Collaborative Innovation Center of High Performance Computing, National University of Defense Technology, Changsha 410073, P. R. China

## Abstract

hTERT, a catalytic component of human telomerase, is undetectable in normal somatic cells but up-regulated in cancer and stem cells where telomere length is maintained by telomerase. Accumulated evidence indicates that hTERT may have noncanonical functions beyond telomerase by regulating the expression of particular genes. However, comprehensive identification of the genes regulated by hTERT is unavailable. In this report, we expressed WT hTERT and hTERTmut which displays dysfunctional catalytic activity, in human U2OS cancer cells and VA-13 immortalized fibroblast cells, both of which lack endogenous hTERT and hTR expression. Changes in gene expression induced by hTERT and hTERT-mut expression were determined by genome-wide RNA-seq and verified by qPCR. Our results showed that hTERT affects different genes in two cell lines, implying that the regulation of gene expression by hTERT is indirect and cell type dependent. Moreover, functional analysis identifies cell adhesion-related genes that have been changed by hTERT in both cell lines. Adhesion experiments revealed that hTERT expression significantly increases cell adhesion. Monolayer wound healing and transwell assays demonstrated increased cell migration upon hTERT expression. These results provide new evidence to support a noncanonical function for hTERT in promoting tumorigenesis.

Telomerase is a reverse transcriptase that adds tandem telomeric sequences to the end of chromosomes^1^,^2^. Telomerase is composed of two main subunits, the catalytic subunit hTERT and the RNA template hTR^3^,^4^. In most human cancers and germ/stem cells, hTERT catalyzes *de novo* repeat addition using hTR as a template sequence, thus preventing telomere shortening caused by the “end-replication problem” and end-processing. In human somatic cells, telomerase is absent and telomeres are progressively shortened until a critical length is reached that triggers cell senescence or apoptosis. More than 80% of tumors express telomerase^3^; activation of hTERT expression is a critical step in carcinogenesis.

Accumulated evidence demonstrates that hTERT has non-canonical functions beyond telomere lengthening. It has been reported that telomerase that lacks extension activity promotes tumorigenesis^5^. Moreover, Artandi’s group found that TERT mutations that lack catalytic activity could induce the proliferation of hair follicle stem cells in transgenic mice, possibly through transcriptional regulation of the Wnt signaling pathway^6^,^7^. TERT has also been found to play roles in apoptosis, DNA damage response, and regulation of gene expression^8^. Ectopic expression of hTERT was able to promote cell proliferation by either upregulating epiregulin or EGFR expression in human cells^9^,^10^,^11^. In cancer cells, overexpression of hTERT enhances the progression of gastric cancer by upregulating Mac-2BP^12^. These studies revealed that hTERT has a variety of functions aside from telomere extension. In particular, these functions involve the up- and down-regulation of some important genes. However, comprehensive understanding of genome-wide gene expression regulated by hTERT remains unclear. Although altered mRNA profiling has been reported in human and mice cells overexpressing the TERT gene, the results are complicated by the fact that increased TERT affects telomere length homeostasis that could interfere with gene transcription. To this end, it is important to study non-canonical hTERT functions in telomerase-deficient cells.

In this report, we overexpressed hTERT in human ALT cancer U2OS cells and immortalized fibroblast cells VA-13, both of which lack endogenous hTR and hTERT expression, thus preventing the influence of changes in telomere length on gene expression. We also overexpressed an hTERT with mutated amino acids that lacked catalytic activity. Comparison of gene expression profiling in cells with and without hTERT expression (or mutant hTERT) rendered the conclusion that hTERT is implicated in the regulation of cell adhesion-related genes. These experiments also demonstrated that hTERT or mutant hTERT overexpression promotes cell migration and transformation.

## Results

### Ectopic expression of hTERT or hTERTmut in different cells show distinct expression profiles

**Figure 1:**
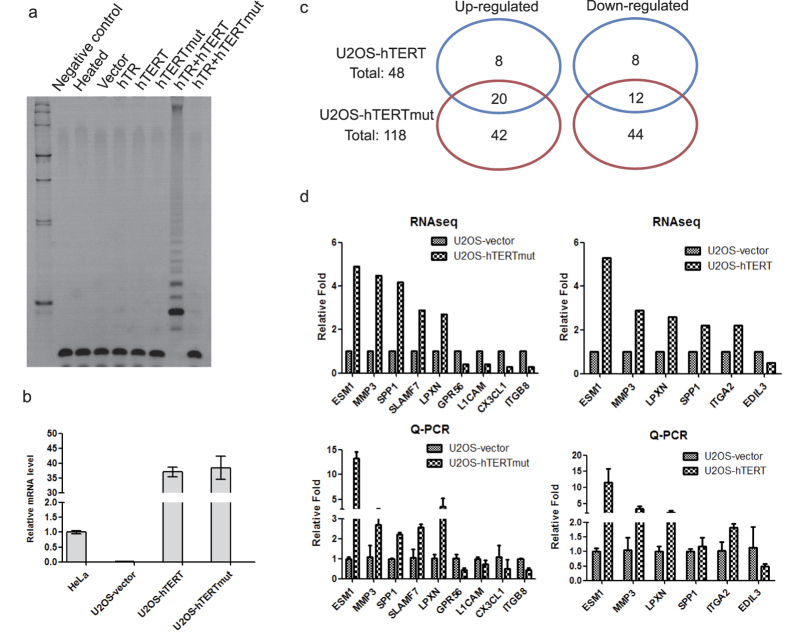
Dysregulated genes in U2OS-hTERT and U2OS-hTERTmut cells. (**a**) Telomerase activity was detected by TRAP assay. Only U2OS cells transfected with both hTERT and hTR showed telomerase activity. (**b**) hTERT and hTERTmut stably overexpressed in U2OS with empty vector as a control. hTERT mRNA levels were quantified by qPCR and normalized to HeLa cells. (**c**) Venn diagrams depicting the genes that were up- and down-regulated by hTERT and hTERTmut in U2OS. The gene expression profiles were determined by RNA-seq as described in the Materials and methods section. (**d**) RNA-seq results were confirmed by qPCR. Several differentially expressed genes were randomly selected in the indicated stable cell lines. RNA-seq and qPCR results are showed in the upper and lower panels, respectively.

Mutant hTERT (hTERTmut) was constructed by substituting the valine and isoleucine residues at positions 710 and 711 with aspartic acid and alanine residues, respectively^13^. TRAP assay showed that human cancer U2OS cells have no detectable telomerase activity. Overexpression of either WT hTERT or hTR alone displayed no telomerase activity, indicating the lack of endogenous expression of hTERT and hTR (Fig. 1a). Telomerase activity was detected in cells co-expressing WT hTERT and hTR, but not in those expressing hTERTmut and hTR, demonstrating the loss of catalytic activity in the hTERTmut (Fig. 1a).

Wild-type hTERT or hTERTmut was stably expressed in U2OS cells. The empty pBabe vector was used as a control (Fig. 1b). To explore changes in gene expression due to hTERT or hTERTmut expression, whole genome gene expression profiles of U2OS-hTERT, U2OS-hTERTmut, and U2OS-vector cell lines were determined by RNA-seq using next-generation sequencing. Differentially expressed genes were sorted with the DESeq package^14^, where transcripts with adjusted p-value < 0.05 (padj) were considered as valid candidates. The results showed that expression of wild-type hTERT resulted in 48 changes compared with the empty vector, including 28 up-regulated and 20 down-regulated genes. The expression of hTERTmut led to 118 changes compared with the vector control, including 62 up-regulated and 56 down-regulated genes (Supplementary Table S1). Thirty two common changes were found, including 20 up-regulated and 12 down-regulated genes (Fig. 1c).

To validate the RNA-seq data, randomly selected genes were subjected to quantitative RT-PCR to determine the change in expression level when hTERT or hTERTmut was expressed (Fig. 1d and Fig. S1). Our results showed that these genes displayed changes in their expression levels similar to the results obtained by RNA-seq, demonstrating that the RNA-seq data were reliable.

**Figure 2:**
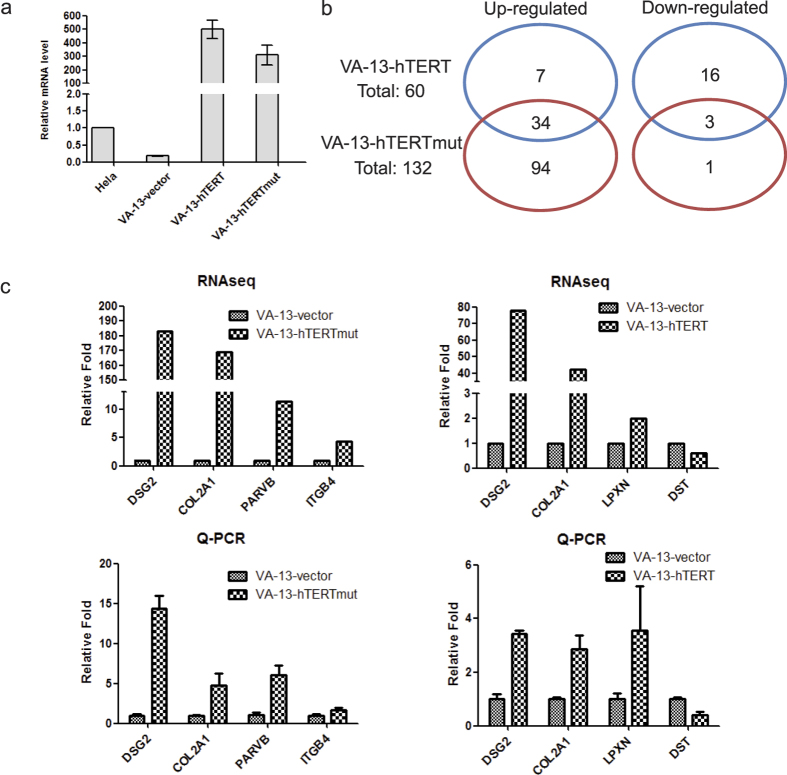
Dysregulated genes in the VA-13-hTERT and VA-13-hTERTmut cells. (**a–c**) Same as (b–d) in Fig. 1 with VA-13 cells instead of U2OS cells.

We then tested whether hTERT or hTERTmut affected gene expression in another cell line, VA-13, an immortalized human fibroblast cell line with no detectable expression of either hTERT or hTR. Wild-type hTERT, hTERTmut, and empty vector were expressed in VA-13 cells and gene expression profiles were obtained by RNA-seq (Fig. 2). Data analysis showed that hTERT expression up-regulated 41 genes and down-regulated 19 genes, while expression of hTERTmut up-regulated 128 genes and down-regulated 4 genes (Supplementary Table S2). There were 37 commonly changed genes on both lists, including 34 up-regulated and 3 down-regulated genes (Fig. 2b). Differently expressed genes identified by RNA-seq were also validated by individual quantitative RT-PCR. The consistent results were obtained that demonstrated the reliability of RNA-seq (Fig. 2c and Fig. S2). Interestingly, only one gene, KRT17, was changed in both U2OS and VA-13 cells. Possible reasons for the lack of overlap between the two cell lines may be that hTERT regulates gene transcription by associating with other modulating factors such as BRG1 and p65^15^,^16^. Different associating factors may be present in U2OS and VA-13 cells that determine which gene hTERT targets.

### Characterization of the functional changes due to hTERT or hTERTmut expression

**Figure 3:**
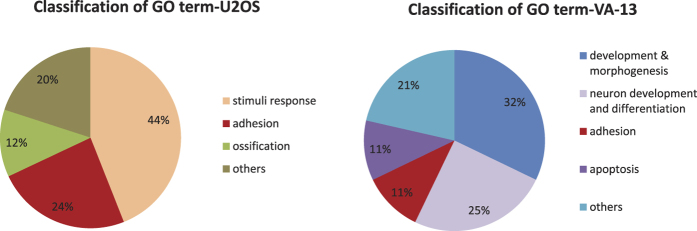
Categorization of commonly regulated hTERT and hTERTmut GO terms. The biological processes commonly enriched in U2OS-hTERT and U2OS-hTERTmut cells were classified into different group according to the related functions.

Although hTERT regulates different genes in U2OS and VA-13 cells, functional similarities between these genes may exist. Thus, we performed gene functional analysis using Gene Ontology (GO)^17^. The biological processes enriched in both hTERT and hTERTmut expressed cells are shown in Table 1 and Table 2 (U2OS and VA-13 cell lines, respectively). The GO terms can be classified into different categories according to their functions (Fig. 3). In U2OS cells, the commonly altered GO terms due to hTERT and hTERTmut expression are involved in stimuli response (44%), adhesion (24%), ossification (12%), and others (20%) (Table 1 and Fig. 3). Stimuli response- and ossification-related genes are consistent with the identity of U2OS as osteosarcoma cells. In VA-13 cells, the GO terms are related to development & morphogenesis (32%), neuron development & differentiation (25%), adhesion (11%), apoptosis (11%), and others (Table 2 and Fig. 3). More than 50% (32% + 25%) of the GO terms are related to development, reflecting the nature of VA-13 cells, which originate from WI38 fibroblasts with stem-like features and have the potential to be reprogrammed into stem cells or directly converted into neuronal cells^18^,^19^,^20^.

### hTERT regulates cell adhesion independent of its telomerase activity

**Figure 4:**
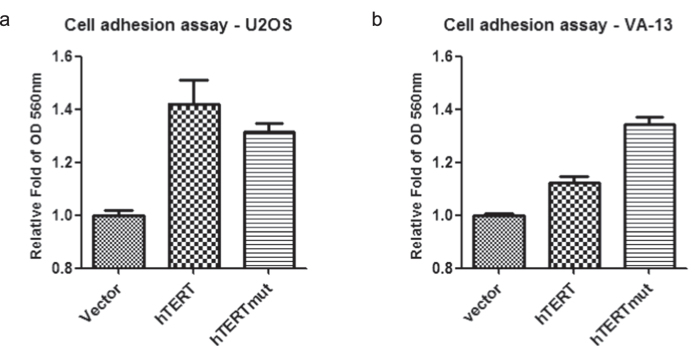
hTERT and hTERTmut expression promoted cell adhesion to the ECM. (**a**) U2OS-vector, U2OS-hTERT, and U2OS-hTERTmut cells were cultured on fibronectin-coated plates for 1 hour and the adherent cells were detected by crystal violet staining. (**b**) VA-13-vector, VA-13-hTERT, and VA-13-hTERTmut cells were cultured on fibronectin-coated plates for 1 hour and adherent cells were detected by crystal violet staining.

Strikingly, cell adhesion-related genes are present in the catalogues from both the U2OS and VA-13 cells expressing hTERT or hTERTmut (Fig. 3, Tables S3 & S4). This result encouraged us to propose that hTERT may function in regulating cell adhesion independent of its role in telomerase activity. To test this hypothesis, we performed cell adhesion assays using fibronectin as a substrate. The results showed that hTERT or hTERTmut expressing cells displayed increased adhesion to the extracellular matrix (ECM) (Fig. 4).

**Figure 5:**
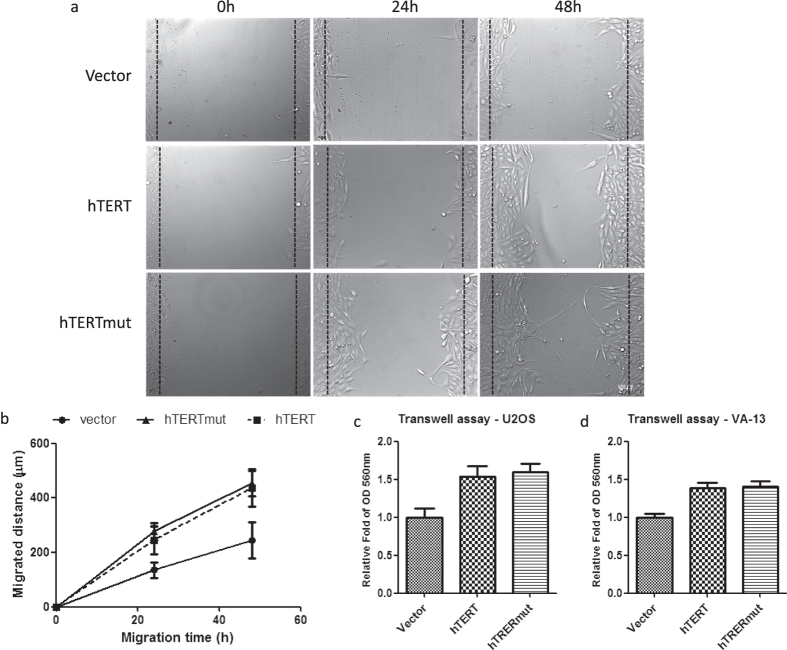
hTERT and hTERTmut expression promoted cell migration. (**a**) hTERT and hTERTmut expression promoted U2OS cell monolayer wound healing. Cells were cultured in a 6-well plate. After scratching a wound, cells were cultured in serum-free medium and photographed at 0, 24, and 48 h. (**b**) Statistical data from the cell migration distances in (**a**). (**c,d**) hTERT and hTERTmut expression promoted U2OS and VA-13 cell migration in a transwell assay.

Cell adhesion is particularly important in tumor progression and metastasis. During metastasis, cell adhesion is down-regulated to detach cells from the primary carcinoma and then up-regulated to attach the same cells to distant tissues^21^. In this context, increased cell adhesion may enhance cell migration. We carried out monolayer wound healing and transwell assays to explore cell migration abilities. Wound closure speeds were significantly increased in both U2OS-hTERT and U2OS-hTERTmut expressed cells compared with control cells (Fig. 5a,b). Consistently, hTERT or hTERTmut expressing cells displayed an elevated ability to traverse a membrane from the serum-free to serum side (10% FBS) in the transwell assay (Fig. 5c,d).

## Discussion

Noncanonical functions of TERT have been previously reported. For example, TERT protein has been shown to be involved in regulating gene expression of the Wnt pathway, thereby promoting hair growth in mice. Moreover, it has also been found that hTERT binds to an NF-κB subunit and directly regulates a subset of NF-κB targeted genes, such as IL-6, TNF-α, and MMPs^15^,^22^. Furthermore, TERT has been implicated in production of small interfering RNAs in a Dicer (also known as DICER1)-dependent manner. Despite the significance of these findings, it is challenging to extend these conclusions to different species or cell types^23^. Our data demonstrate that hTERT regulates different genes in different cells. Similar results have been reported previously^6^,^9^,^10^,^11^,^12^. For example, it was found that 172 genes displayed changes in expression in hTERT-immortalized BJ fibroblasts^10^. In contrast, in telomerase-immortalized bovine adrenocortical cell, 284 genes displayed changes in expression^11^. However, overlapping genes that exhibited changes in their expression between the two cell lines were very rare. These results and ours lead to the conclusion that hTERT regulates gene transcription in a cell type-dependent manner. A possible explanation is that regulation of gene expression by hTERT is indirect and mediated by co-factors that might be different in different cell types. In support of this hypothesis, hTERT has been reported to associate with BRG1, p65, and RMRP to modulate the transcription of genes related to the Wnt, NF-κB, and siRNA pathways, respectively^15^,^16^,^22^,^24^. Given that these genes might be differentially expressed in different cell lines, the downstream genes regulated by hTERT might also be different.

Even though hTERT expression mostly affected different genes in U2OS and VA-13 cells, a similar change in function was indeed found. Gene functional analysis identified a significant number of genes that are potentially involved in cell adhesion and migration. Of them many genes have been previously demonstrated to affect cell motion, EMT, cell invasion and cancer metastasis through regulating cell adhesion. For example, PDPN, SPP1, BARX2 and MMPs genes are reported to promote cell adhesion to ECM^25^,^26^,^27^,^28^,^29^; DSG2 facilitates the formation of structure that mediates cell-cell adhesion^30^; IL-8 enhances colon cancer cell migration by activating the expression of a disintegrin and metalloprotease (ADAM), a enzyme required for cell motion^31^; and ARHGDIB induces EMT, thus promoting cell invasion and cancer metastasis^32^,^33^.

Besides up-regulated genes, we also found that several genes are down-regulated by hTERT or hTERTmut. For instance, COL3A1 and GPR56 are reported to suppress the migration of neuron cells^34^; their down-regulation is expected to promote cell migration. Moreover, KRT17 is the only gene identified that is upregulated by expression of hTERT or hTERTmut in both U2OS and VA-13. It has been found that KRT17 promotes cell adhesion by activating AKT/PKB signaling pathway^35^.

Because hTERTmut and WT hTERT have the same phenotype, we concluded that the function of hTERT in promoting adhesion and cell migration was independent of telomerase activity. It was previously reported that TERT transgenic mice were more susceptible to develop skin tumors upon chemical carcinogenesis compared with normal mice^36^. Consistently, both wild-type hTERT and mutant hTERT without catalytic activity were reported to promote tumorigenesis in human cells^5^, and knockdown of endogenous hTERT could inhibit tumorigenicity in human HCT116 cells^37^. The mechanism for enhanced tumorigenesis by hTERT is complicated and^37^ might involve multiple events, including cell proliferation, anti-apoptosis, energy metabolism, tolerance to chromosomal instabilities, etc^38^,^39^,^40^,^41^,^42^,^43^. Many of these events might be related to telomere length homeostasis. In our experiments, U2OS are typical cancer cells that use the alternative lengthening of telomeres (ALT) pathway to maintain their telomere length, while VA-13 are immortalized cells that stem from human normal fibroblast cells. Our results indicated that the promotion of cell adhesion and migration may be a common function of hTERT in human cells. Our results also showed that although the genes are different in the two cells, some of them are involved in the formation of extracellular matrix (ECM) and matrix metalloproteinases (MMPs), a group of proteases responsible for ECM proteolysis. This provides a clue for further study regarding the regulatory mechanism underlying the increased cell adhesion and migration by hTERT.

## Materials and Methods

### Cell culture, vectors, and transfections

U2OS and VA-13 cells were cultivated in Dulbecco’s Modified Eagle Medium with 10% fetal bovine serum, 100 U/ml penicillin, and 100 μg/ml streptomycin. To generate hTERT or hTERTmut stably overexpressing cell lines, 293 T cells were transfected with pBabe-hTERT, pBabe-hTERTmut, or empty pBabe plasmids and the retroviral packaging plasmids pCMV-VSV.G and pCMV-Gag-Pol (Addgene) using calcium phosphate precipitation. The viral supernatants were collected 72 h after transfection, ultracentrifugated at 40,000 rpm for 2 h at 4 °C, and then used to infect U2OS and VA-13 cells. Forty-eight hours later, cells were selected with 2 μg/ml puromycin for 3 days, and then the retained cells were cultured in 1 μg/ml puromycin to produce a polyclonal cell population.

### Cell adhesion assay

A 96-well plate was coated with 2.5 μg/ml human fibronectin (Millipore, CA) for 2 h at room temperature. Cells were seeded into a 96-well plate at a density of 4 × 10^4^ cells/well and cultured for 1 h at 37 °C in a CO_2_ incubator. The cells were then rinsed three times with 10% formalin and stained with crystal violet for 5 min at room temperature. Then, the cells were washed three times with ddH2O and dissolved with 100 μl 33% acetic acid. The absorbance was detected at 560 nm on a Synergy H1 Multi-Mode Reader (BioTek).

### Cell migration assay

Cell migration was measured by the transwell method. Briefly, cells were serum-deprived for 4 h and then seeded into Transwell Permeable Supports (Corning, NY), which were pre-equilibrated with serum-free DMEM for 1 h. For each group, 1 × 10^5^ cells/insert were seeded into the upper compartment of the transwell in 100 μl serum-free DMEM. The insert was put into a 24-well plate that contained 600 μl of DMEM with 10% FBS. After 24 h of culture, the upper surface of the filter was erased with a cotton swab. Then, 10% formalin was added to the lower chamber for 10 min and subsequently stained with crystal violet for 5 min at room temperature. The insert was then washed three times with ddH2O and the crystal violet stained cells dissolved with 500 μl of 33% acetic acid. The absorbance was detected at 560 nm on a Synergy H1 Multi-Mode Reader (BioTek).

### Monolayer wound healing assay

Cells were seeded into a 6-well plate at a density of 7 × 10^5^ cells/well and cultured until confluent. A yellow pipette tip was used to make a straight scratch, which simulated a wound, and then the media was replaced with serum-free DMEM. Images were taken at 0, 24, and 48 h after scratching using a 10x objective.

### Sample preparation and sequencing

Total RNA was extracted using the Trizol reagent (Takara). A total of 3 μg RNA per sample was used as input material for the RNA sample preparations. Sequencing libraries were generated using a NEBNext Ultra RNA Library Prep Kit for Illumina (NEB, USA) according to the manufacturer’s recommendations. Index codes were added to attribute sequences to each sample. Briefly, mRNA was purified from total RNA using poly-T oligo-attached magnetic beads. Fragmentation was carried out using divalent cations under elevated temperature in NEBNext First Strand Synthesis Reaction Buffer (5X). First strand cDNA was synthesized using random hexamer primers and M-MuLV Reverse Transcriptase (RNase H^−^). Second strand cDNA synthesis was subsequently performed using DNA polymerase I and RNase H. The remaining overhangs were converted into blunt ends with exonuclease/polymerase activities. After adenylation of the DNA fragment 3′ ends, NEBNext Adaptors with hairpin loop structures were ligated to prepare for hybridization. The library fragments were purified with an AMPure XP system (Beckman Coulter, Beverly, USA) to select cDNA fragments that were preferentially 150–200 bp in length. Then, 3 μl USER Enzyme (NEB, USA) was added to the size-selected, adaptor-ligated cDNAs at 37 °C for 15 min followed by 5 min at 95 °C before PCR. PCR was performed with Phusion High-Fidelity DNA polymerase, Universal PCR primers, and index (X) primers. Finally, PCR products were purified (AMPure XP system) and the library quality was assessed on an Agilent Bioanalyzer 2100 system. The clustering of the index-coded samples was performed on a cBot Cluster Generation System using a TruSeq SR Cluster Kit v3-cBot-HS (Illumia) according to the manufacturer’s instructions. Following cluster generation, the library preparations were sequenced on an Illumina Hiseq 2000 platform and 100 bp paired reads were generated.

### Data analysis

All of the RNA-Seq reads were first checked with FastQC (http://www.bioinformatics.babraham.ac.uk/projects/fastqc/). Using homemade Java and GNUBash scripts, unpaired reads and those containing low PHRED score (<Q20) bases, Ns, or no adaptors were removed from the raw reads. The remaining high-quality 100 bp paired-end reads were then mapped to the UCSC build hg19 human genome with Tophat2 (Version 2.0.2)^44^ in paired-end mode. Reads that uniquely aligned were used for further analysis. The Htseq-count script (Version 0.6.0)^3^ was employed to assign the uniquely mapped reads to the genes. To obtain the expression level of each gene in different samples, fragments per kilobase per million sequenced reads (FPKM) was manually calculated per Trapnell C *et al.*^45^. Differential expression analysis was performed using the DESeq (version 1.20) package^14^ and genes with adjusted p-values smaller than 0.05 were considered to be differentially expressed.

## Additional Information

**Accession codes:** The raw data of RNAseq have been submitted to GEO database. The GEO accession number is GSE73277.

**How to cite this article**: Liu, H. *et al.* hTERT promotes cell adhesion and migration independent of telomerase activity. *Sci. Rep.* **6**, 22886; doi: 10.1038/srep22886 (2016).

## Supplementary Material

Supplementary Information

## Figures and Tables

**Table 1:** hTERT and hTERTmut co-related biology process in U2OS.

Biology Process	U2OS-hTERT	U2OS-hTERTmut
	PValue	PValue
GO:0010033~response to organic substance	0.0004	0.0029
**GO:0030155~regulation of cell adhesion**	0.0004	0.0003
GO:0007584~response to nutrient	0.0004	0.0003
GO:0033273~response to vitamin	0.0006	0.0010
GO:0031667~response to nutrient levels	0.0016	0.0020
**GO:0032963~collagen metabolic process**	0.0024	0.0008
GO:0009991~response to extracellular stimulus	0.0024	0.0035
GO:0044259~multicellular organismal macromolecule metabolic process	0.0029	0.0011
GO:0009719~response to endogenous stimulus	0.0036	0.0055
GO:0044236~multicellular organismal metabolic process	0.0041	0.0019
GO:0001649~osteoblast differentiation	0.0052	0.0000
**GO:0010810~regulation of cell-substrate adhesion**	0.0063	0.0375
**GO:0007155~cell adhesion**	0.0084	0.0001
**GO:0022610~biological adhesion**	0.0085	0.0001
GO:0009612~response to mechanical stimulus	0.0092	0.0061
**GO:0045785~positive regulation of cell adhesion**	0.0105	0.0074
GO:0048729~tissue morphogenesis	0.0111	0.0320
GO:0040012~regulation of locomotion	0.0132	0.0391
GO:0009725~response to hormone stimulus	0.0144	0.0111
GO:0009628~response to abiotic stimulus	0.0145	0.0359
GO:0006952~defense response	0.0200	0.0007
GO:0060341~regulation of cellular localization	0.0259	0.0248
GO:0001503~ossification	0.0355	0.0001
GO:0014070~response to organic cyclic substance	0.0390	0.0468
GO:0060348~bone development	0.0401	0.0002

**Table 2:** hTERT and hTERTmut co-related biology process in VA-13.

Biology Process	VA-13-hTERT	VA-13-hTERTmut
	PValue	PValue
GO:0030182~neuron differentiation	0.0003	0.0000
GO:0048666~neuron development	0.0005	0.0001
**GO:0030198~extracellular matrix organization**	0.0038	0.0460
**GO:0007155~cell adhesion**	0.0047	0.0894
**GO:0022610~biological adhesion**	0.0047	0.0900
GO:0022604~regulation of cell morphogenesis	0.0072	0.0005
GO:0031175~neuron projection development	0.0073	0.0036
GO:0042981~regulation of apoptosis	0.0098	0.0446
GO:0043067~regulation of programmed cell death	0.0103	0.0473
GO:0010941~regulation of cell death	0.0105	0.0483
GO:0030705~cytoskeleton-dependent intracellular transport	0.0108	0.0074
GO:0008360~regulation of cell shape	0.0116	0.0082
GO:0006928~cell motion	0.0138	0.0299
GO:0001501~skeletal system development	0.0155	0.0032
GO:0060351~cartilage development involved in endochondral bone morphogenesis	0.0181	0.0453
GO:0007409~axonogenesis	0.0205	0.0166
GO:0030030~cell projection organization	0.0247	0.0074
GO:0048667~cell morphogenesis involved in neuron differentiation	0.0252	0.0055
GO:0048812~neuron projection morphogenesis	0.0265	0.0242
GO:0042127~regulation of cell proliferation	0.0300	0.0010
GO:0008284~positive regulation of cell proliferation	0.0359	0.0013
GO:0035107~appendage morphogenesis	0.0361	0.0000
GO:0035108~limb morphogenesis	0.0361	0.0000
GO:0000904~cell morphogenesis involved in differentiation	0.0374	0.0113
GO:0048858~cell projection morphogenesis	0.0378	0.0406
GO:0048736~appendage development	0.0388	0.0000
GO:0060173~limb development	0.0388	0.0000
GO:0001944~vasculature development	0.0401	0.0442
GO:0043627~response to estrogen stimulus	0.0402	0.0087
GO:0007411~axon guidance	0.0416	0.0093
GO:0032990~cell part morphogenesis	0.0421	0.0474
