# Supplementary material for: hTERT promotes cell adhesion and migration independent of telomerase activity
Source: Sci Rep. 2016 Mar 14;6:22886. doi: 10.1038/srep22886 (PMC4789728; doi:10.1038/srep22886)
Supplement: Supplementary Information [file srep22886-s1.pdf]

Supplementary information

hTERT promotes cell adhesion and migration independent of telomerase activity

Haiying Liu[1,2,3], Qianqian Liu[1,2,3], Yuanlong Ge[1,2], Qi Zhao[1], Xiaohui Zheng[1,2], Yong Zhao[1,2] *

[1] Key Laboratory of Gene Engineering of the Ministry of Education, School of Life Sciences, Sun Yat-sen University, Guangzhou 510006 P. R. China;

[2]Collaborative Innovation Center of High Performance Computing, National University of Defense Technology, Changsha 410073, P. R. China;

[3] Those authors contributed equally to this work.

* To whom correspondence should be addressed:

YZ, E-mail: zhaoy82@mail.sysu.edu.cn, Tel: 86-203-994-3401, Fax: 86-203-994-3778

Figure S1

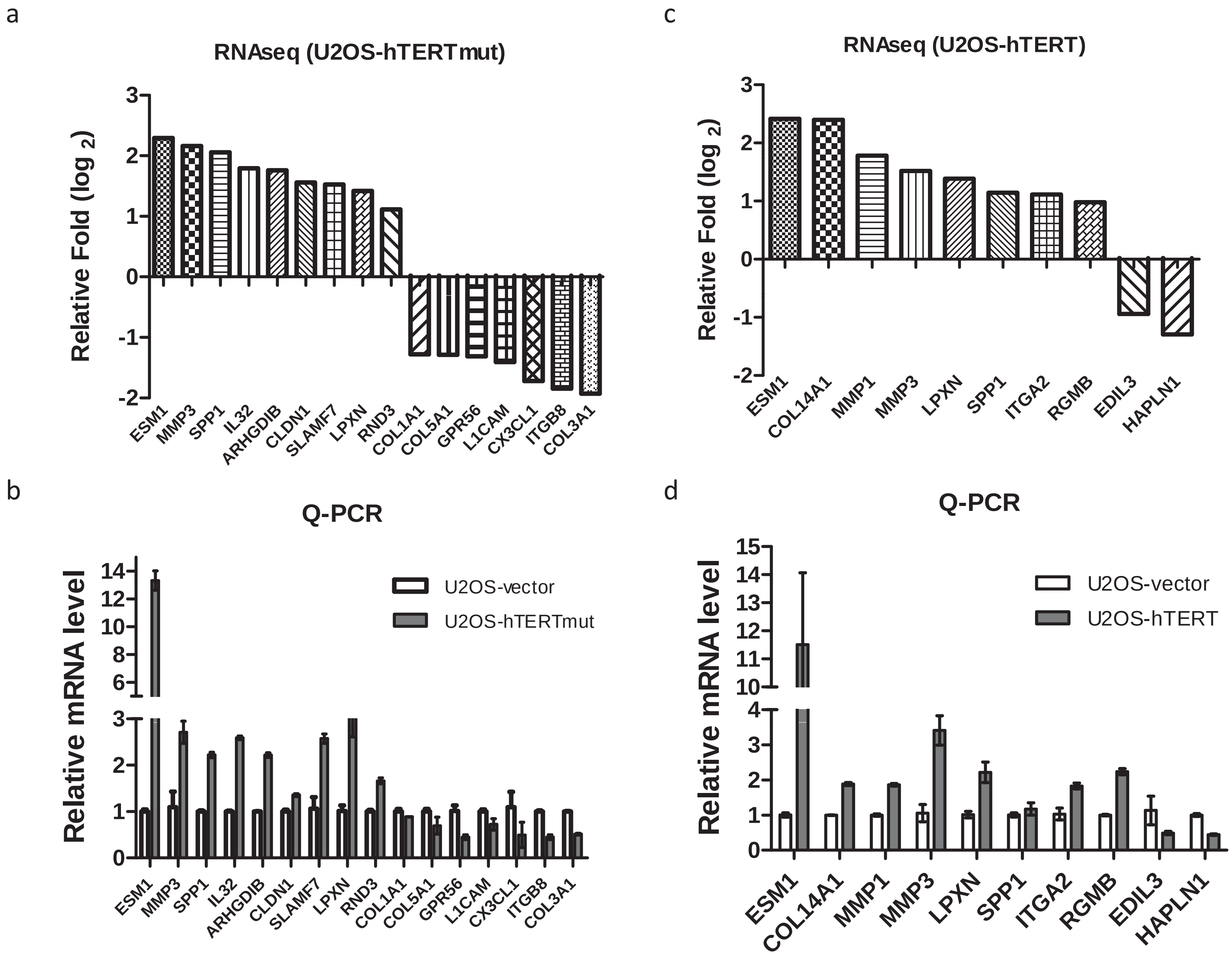
a
RNAseq (U2OS-hTERTmut)
Relative Fold (log 2)
3
2
1
0
-1
-2
ESM1
MMP3
SPP1
IL32
ARHGDIB
CLDN1
SLAMF7
LPXN
RND3
COL1A1
COL5A1
GPR56
L1CAM
CX3CL1
ITGB8
COL3A1
c
RNAseq (U2OS-hTERT)
Relative Fold (log 2)
3
2
1
0
-1
-2
ESM1
COL14A1
MMP1
MMP3
LPXN
SPP1
ITGA2
RGMB
EDIL3
HAPLN1
b
Q-PCR
Relative mRNA level
14
12
10
8
6
3
2
1
0
U2OS-vector
U2OS-hTERTmut
ESM1
MMP3
SPP1
IL32
ARHGDIB
CLDN1
SLAMF7
LPXN
RND3
COL1A1
COL5A1
GPR56
L1CAM
CX3CL1
ITGB8
COL3A1
d
Q-PCR
Relative mRNA level
15
14
13
12
11
10
4
3
2
1
0
U2OS-vector
U2OS-hTERT
ESM1
COL14A1
MMP1
MMP3
LPXN
SPP1
ITGA2
RGMB
EDIL3
HAPLN1

Figure S2

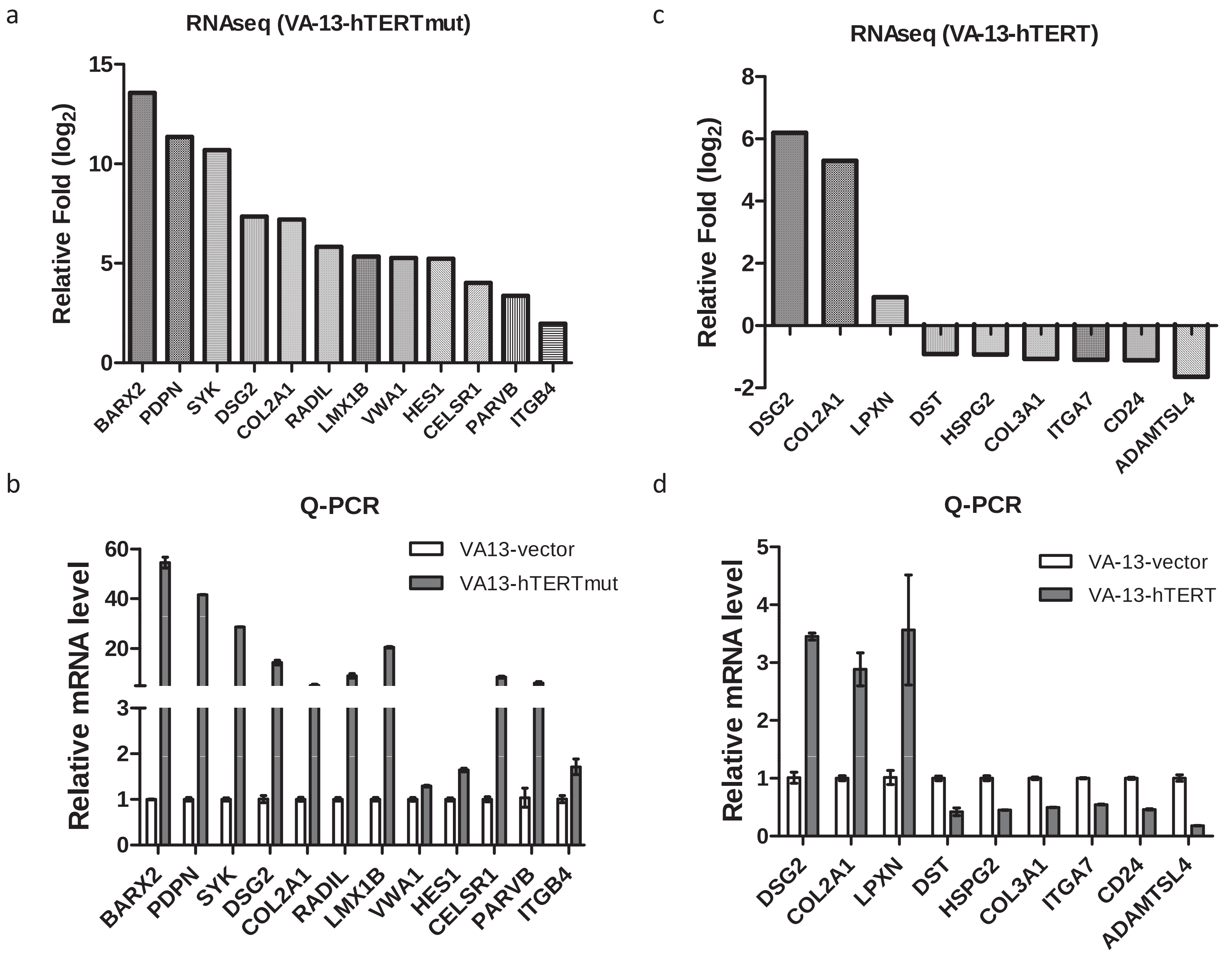
a
RNAseq (VA-13-hTERTmut)
Relative Fold (log2)
15
10
5
0
BARX2
PDPN
SYK
DSG2
COL2A1
RADIL
LMX1B
VWA1
HES1
CELSR1
PARVB
ITGB4
c
RNAseq (VA-13-hTERT)
Relative Fold (log2)
8
6
4
2
0
-2
DSG2
COL2A1
LPXN
DST
HSPG2
COL3A1
ITGA7
CD24
ADAMTSL4
b
Q-PCR
VA13-vector
VA13-hTERTmut
Relative mRNA level
60
40
20
3
2
1
0
BARX2
PDPN
SYK
DSG2
COL2A1
RADIL
LMX1B
VWA1
HES1
CELSR1
PARVB
ITGB4
d
Q-PCR
VA-13-vector
VA-13-hTERT
Relative mRNA level
5
4
3
2
1
0
DSG2
COL2A1
LPXN
DST
HSPG2
COL3A1
ITGA7
CD24
ADAMTSL4

Figure legends

Figure S1: RNA-seq results were confirmed by qPCR. 16 (a and b) and 10 (c and d) differently expressed genes were selected from U2OS cells expressing hTERTmut and hTERT, respectively.

Figure S2: RNA-seq results were confirmed by qPCR. 12 (a and b) and 9 (c and d) differentially expressed genes were selected from VA13 cells expressing hTERTmut and hTERT, respectively.

Table S1 Dysregulated genes in U2OS cells

U2OS+hTERT			U2OS+hTERTmut		
**Gene**	**log2FoldChange**	**padj**	**Gene**	**log2FoldChange**	**padj**
TERT	9.924	0.000	TERT	10.098	0.000
LOC100505875	5.174	0.001	MSR1	9.873	0.005
TNFSF8	3.763	0.000	LOC100505875	6.564	0.000
IGFN1	2.953	0.045	TNFSF8	4.082	0.000
KYNU	2.936	0.000	PRR9	4.028	0.027
IL8	2.514	0.000	BCL2A1	3.745	0.020
ESM1	2.413	0.000	BEST3	3.702	0.007
COL14A1	2.398	0.045	SLC28A3	3.566	0.037
MYCT1	2.251	0.002	IL8	3.198	0.000
IL13RA2	2.160	0.000	MYCT1	3.117	0.000
MMP1	1.782	0.011	IGFN1	3.111	0.047
LINC00520	1.668	0.007	TRAF1	3.023	0.039
VEPH1	1.637	0.036	KYNU	2.962	0.000
MMP3	1.518	0.000	IL13RA2	2.695	0.000
RGS4	1.470	0.001	MYOZ2	2.688	0.026
FGF5	1.394	0.000	GAGE1	2.587	0.001
LPXN	1.387	0.001	ESM1	2.295	0.000
SP140	1.360	0.028	LOC100288077	2.278	0.015
LOC152225	1.292	0.004	HSPA6	2.271	0.004
PTX3	1.221	0.046	LINC00520	2.185	0.000
ADAMTS6	1.168	0.002	MMP3	2.160	0.000
SCG5	1.168	0.002	SPP1	2.055	0.000
PAPPA2	1.149	0.039	VEPH1	1.923	0.024
ANTXR2	1.144	0.002	NLRP1	1.910	0.003
SPP1	1.142	0.012	SP140	1.829	0.001
ITGA2	1.115	0.001	IL32	1.795	0.007
ELTD1	1.091	0.027	TMEM71	1.791	0.032
RGMB	0.983	0.037	BIRC3	1.773	0.029
HMGA2	0.899	0.017	ARHGDIB	1.761	0.001
HSPB1	-0.811	0.049	SRGN	1.722	0.000
FADS2	-0.858	0.028	DUSP5	1.662	0.001
COL1A1	-0.859	0.026	TNFAIP3	1.624	0.009
KRT17	-0.893	0.017	UCA1	1.612	0.005
CSDC2	-0.922	0.046	LCP1	1.604	0.000
EDIL3	-0.941	0.020	CLDN1	1.560	0.026
FAM19A5	-0.999	0.045	PAPPA2	1.529	0.002
MN1	-1.004	0.035	SLAMF7	1.528	0.013
CREB3L1	-1.106	0.009	RIMS2	1.525	0.019
TNNT2	-1.183	0.014	FGF5	1.473	0.000
PIEZO2	-1.211	0.046	LPXN	1.418	0.007
DRD1	-1.220	0.048	CD163L1	1.406	0.002

HAPLN1	-1.293	0.000	KISS1	1.385	0.000
LBH	-1.302	0.000	KRT34	1.368	0.003
FOSB	-1.360	0.001	SCG5	1.352	0.001
BMP4	-1.400	0.000	TMEM154	1.332	0.026
FLJ22184	-1.574	0.026	HMOX1	1.326	0.001
CXCR7	-1.589	0.020	LOC152225	1.303	0.021
EDN2	-1.630	0.007	HKDC1	1.300	0.042
TNNC1	-1.726	0.001	MAFF	1.282	0.009
			GLIPR1	1.187	0.024
			CSTA	1.146	0.021
			HDAC9	1.144	0.017
			ANTXR2	1.133	0.009
			RND3	1.114	0.004
			EFR3B	1.083	0.009
			ADAMTS6	1.061	0.033
			VGLL3	1.030	0.027
			LOX	1.015	0.009
			PPP1R15A	0.972	0.023
			LIMCH1	0.931	0.021
			TXNRD1	0.929	0.026
			SYTL3	0.928	0.044
			SLC7A11	0.883	0.032
			RHOBTB3	-0.917	0.027
			OSBPL10	-0.931	0.037
			FADS2	-0.938	0.023
			ENC1	-0.962	0.021
			LOXL4	-0.964	0.047
			COL11A1	-0.969	0.038
			ODZ2	-1.001	0.009
			COL6A3	-1.037	0.008
			SERPINA1	-1.112	0.013
			CYP24A1	-1.132	0.010
			PMEPA1	-1.142	0.001
			SEMA6B	-1.157	0.006
			FAM19A5	-1.167	0.026
			TUBA1A	-1.174	0.004
			SOX4	-1.244	0.002
			ZNF608	-1.260	0.004
			COL1A1	-1.276	0.000
			COL5A1	-1.285	0.000
			GPR56	-1.309	0.032
			RBP1	-1.315	0.008
			L1CAM	-1.408	0.000
			SLIT3	-1.454	0.044

CRISPLD1	-1.482	0.015
AXIN2	-1.487	0.005
MN1	-1.522	0.000
ARL4C	-1.529	0.000
TRIB2	-1.531	0.000
C9orf84	-1.543	0.018
TNNC1	-1.543	0.029
COL9A2	-1.568	0.026
RUNX2	-1.590	0.023
LRRN1	-1.609	0.000
BMP4	-1.617	0.000
SLCO2A1	-1.662	0.005
CX3CL1	-1.717	0.032
HR	-1.742	0.000
EDIL3	-1.752	0.000
KRT17	-1.826	0.000
ITGB8	-1.843	0.000
GUCY1A2	-1.888	0.000
DBP	-1.921	0.014
COL3A1	-1.929	0.025
LBH	-1.934	0.000
HAS2	-2.011	0.006
SOX2	-2.128	0.013
KRT14	-2.233	0.003
RNF43	-2.233	0.003
CXCR7	-2.308	0.003
PODNL1	-2.370	0.024
HSD17B3	-2.478	0.001
OLFML2A	-2.487	0.001
IGFBP5	-2.507	0.000
CXCL14	-2.568	0.003
HAPLN1	-2.643	0.000
DRD1	-2.648	0.000
SOSTDC1	-3.197	0.032

Table S2 Dysregulated genes in VA-13 cells

VA-13-hTERT			VA-13+hTERTmut		
Gene	log2FoldChange	padj	Gene	log2FoldChange	padj
CA2	15.074	0.000	CA2	16.844	0.000
GAL	13.584	0.023	CHCHD10	16.094	0.000
CHCHD10	13.582	0.028	GAL	15.306	0.000
HOXD13	13.386	0.000	HOXD13	14.994	0.000
PPP1R14C	12.187	0.041	PCSK1N	14.821	0.000
TUBB4A	12.100	0.011	TUBB4A	14.398	0.000
CDX2	11.957	0.034	CHCHD5	14.350	0.047
PALM	10.814	0.023	IRS4	14.101	0.000
IRS4	10.789	0.028	RPP25	13.891	0.000
DSP	10.765	0.000	PPP1R14C	13.841	0.001
MST4	10.706	0.001	HMX1	13.833	0.000
HS6ST2	10.053	0.028	CDX2	13.791	0.000
TERT	10.037	0.000	HAND1	13.708	0.004
MYH14	9.583	0.000	C1orf233	13.632	0.002
SLAIN1	9.304	0.041	C10orf125	13.618	0.012
ADD2	9.010	0.016	GLB1L2	13.564	0.000
DSG2	6.198	0.000	BARX2	13.563	0.005
WNK2	5.571	0.002	HOXD10	13.154	0.016
EN2	5.475	0.005	INA	13.135	0.000
HOXA10	5.470	0.005	SELENBP1	12.973	0.018
GRB10	5.314	0.009	COLEC12	12.700	0.002
TMEM98	5.304	0.013	PALM	12.548	0.000
COL2A1	5.299	0.011	B4GALNT3	12.320	0.004
CCDC8	5.229	0.019	PNMAL1	12.227	0.000
DPYSL5	5.195	0.000	WSCD1	12.047	0.004
PHYHD1	5.182	0.022	EMILIN3	11.992	0.018
CXorf57	5.047	0.038	MATK	11.954	0.001
NUP210	4.537	0.000	GPRC5C	11.890	0.014
EEF1A2	4.495	0.000	DSP	11.785	0.000
KIF1A	4.479	0.012	ZIC5	11.711	0.035
FLT4	4.396	0.020	CAMSAP3	11.448	0.000
CKB	4.004	0.000	PDPN	11.351	0.003
NTN1	3.958	0.022	MYH14	11.302	0.000
PCSK6	3.860	0.029	HS6ST2	11.231	0.006
HEY1	2.603	0.001	MST4	11.193	0.008
MMP1	2.035	0.034	LARGE	11.061	0.012
MT2A	1.112	0.002	ADD2	10.759	0.000
MT1E	0.921	0.004	GPC3	10.751	0.012
LPXN	0.916	0.005	SYK	10.691	0.000
G0S2	0.911	0.005	SLAIN1	10.526	0.008

ETV4	0.865	0.022	SLC6A15	10.480	0.015
SPHK1	0.824	0.019	TERT	10.469	0.000
HEG1	-0.886	0.041	AUTS2	10.247	0.012
DST	-0.909	0.028	KCNQ2	10.049	0.012
HSPG2	-0.932	0.022	RGMA	9.914	0.016
MDGA1	-1.030	0.036	CELF2	9.056	0.047
COL3A1	-1.074	0.013	FGFR2	8.988	0.011
ITGA7	-1.098	0.009	HOXA10	7.423	0.000
CD24	-1.114	0.001	DSG2	7.355	0.000
NTN4	-1.120	0.023	TMEM98	7.298	0.000
MEG3	-1.186	0.001	COL2A1	7.209	0.000
MATN2	-1.300	0.001	EN2	7.201	0.000
NEAT1	-1.350	0.000	WNK2	7.059	0.000
MALAT1	-1.485	0.000	CCDC8	7.047	0.000
PDGFRL	-1.506	0.046	DPYSL5	7.017	0.000
ADAMTSL4	-1.648	0.000	HOXD11	6.977	0.000
LMOD1	-1.707	0.007	EEF1A2	6.856	0.000
SCARNA9	-1.867	0.008	DBNDD1	6.812	0.000
ALPP	-1.971	0.000	RNF130	6.678	0.001
EGR1	-2.135	0.000	KIF1A	6.645	0.000
KRT17	-2.148	0.000	GRB10	6.510	0.001
			PHYHD1	6.509	0.002
			FLT4	6.332	0.000
			CKB	6.248	0.000
			GPC4	6.232	0.008
			KIF26A	6.204	0.000
			NUP210	6.172	0.000
			MSI1	6.150	0.012
			CXorf57	6.108	0.012
			ALX4	6.070	0.015
			NTN1	6.070	0.000
			GNG7	6.039	0.018
			MAF	5.979	0.020
			LINGO1	5.878	0.033
			RADIL	5.829	0.039
			RAB4A	5.543	0.004
			BRSK2	5.509	0.004
			ASS1	5.355	0.000
			LMX1B	5.344	0.010
			VWA1	5.265	0.015
			HES1	5.229	0.002
			LRRC61	5.223	0.018
			B4GALNT4	5.221	0.018
			PCSK6	5.209	0.002

DIRAS1	5.194	0.000
LDOC1	5.184	0.002
CACNA1H	5.144	0.003
HTATIP2	5.112	0.029
PRAME	4.986	0.047
IGFBP2	4.949	0.000
BMP7	4.914	0.000
HOMER2	4.886	0.012
EPB41L3	4.797	0.018
PRKCZ	4.714	0.025
SMOC1	4.668	0.008
CHD7	4.623	0.010
UNC5B	4.548	0.004
NOTCH3	4.428	0.009
JPH3	4.415	0.027
NEFL	4.371	0.033
GPX3	4.364	0.012
FABP5	4.343	0.038
ZCCHC12	4.176	0.000
MAPK8IP2	4.136	0.000
MYO1D	4.135	0.016
RHOU	4.086	0.019
CELSR1	4.013	0.001
SEMA6A	3.835	0.018
ALDH1A2	3.809	0.020
IGSF3	3.778	0.014
HEY1	3.734	0.000
RIMS4	3.651	0.001
SULF2	3.571	0.004
ACSS1	3.547	0.047
PARVB	3.367	0.033
ISYNA1	3.253	0.000
DMKN	3.185	0.000
DUSP9	3.048	0.023
STXBP2	2.935	0.029
MDK	2.909	0.004
SALL2	2.798	0.011
STMN3	2.770	0.028
SPINT2	2.401	0.003
H2AFY2	2.394	0.011
TLE3	2.245	0.003
ITGB4	1.970	0.018
SHISA2	1.902	0.010
GLUL	1.480	0.010

	C19orf63	1.426	0.017
	NEAT1	-1.530	0.031
	F3	-1.600	0.019
	KRT17	-1.616	0.029
	MALAT1	-2.039	0.001

Table S3 Genes related to cell adhesion in U2OS

Biology Process	Genes	
	U2OS-hTERT	U2OS-hTERTmut
GO:0030155~regulation of cell adhesion	IL8, ITGA2, EDIL3, COL1A1, SPP1	IL8, L1CAM, EDIL3, COL1A1, CX3CL1, SPP1, ARHGDIB
GO:0032963~collagen metabolic process	COL1A1, MMP3, MMP1	COL3A1, COL1A1, MMP3, COL5A1
GO:0010810~regulation of cell-substrate adhesion	EDIL3, COL1A1, SPP1	EDIL3, COL1A1, SPP1
GO:0007155~cell adhesion	HAPLN1, RGMB, LPXN, COL14A1, ITGA2, EDIL3, SPP1	HAPLN1, COL3A1, IL32, L1CAM, CX3CL1, SLAMF7, EDIL3, COL5A1, RND3, LPXN, ITGB8, COL6A3, GPR56, CLDN1, COL11A1, SPP1
GO:0022610~biological adhesion	HAPLN1, RGMB, LPXN, COL14A1, ITGA2, EDIL3, SPP1	HAPLN1, COL3A1, IL32, L1CAM, CX3CL1, SLAMF7, EDIL3, COL5A1, RND3, LPXN, ITGB8, COL6A3, GPR56, CLDN1, COL11A1, SPP1
GO:0045785~positive regulation of cell adhesion	ITGA2, EDIL3, SPP1	L1CAM, EDIL3, CX3CL1, SPP1

Table S4 Genes related to cell adhesion in VA-13

Biology Process	Genes	
	VA-13-hTERT	VA-13-hTERTmut
GO:0030198~extracellular matrix organization	ADAMTSL4, COL3A1, HSPG2, COL2A1	LMX1B, SMOC1, COL2A1, VWA1
GO:0007155~cell adhesion	LPXN, DSG2, COL3A1, ITGA7, HSPG2, COL2A1, CD24, DST	HES1, BARX2, DSG2, PDPN, ITGB4, COL2A1, CELSR1, RADIL, PARVB, SYK
GO:0022610~biological adhesion	LPXN, DSG2, COL3A1, ITGA7, HSPG2, COL2A1, CD24, DST	HES1, BARX2, DSG2, PDPN, ITGB4, COL2A1, CELSR1, RADIL, PARVB, SYK
